# A roadmap of priority evidence gaps for the co-implementation of malaria vaccines and perennial malaria chemoprevention

**DOI:** 10.1186/s12936-025-05347-0

**Published:** 2025-04-17

**Authors:** Jane Grant, Perennial Malaria Chemoprevention (PMC) community of practice sub-group on the co-implementation of malaria vaccines and PMC, Jane Grant, Adebanjo Adegbola, Adrian V. S. Hill, Akindeh Nji, Ally Olotu, Brian Greenwood, Charlotte Eddis, Daniel Chandramohan, Dominique Bomba, Farba Faye, Henry Ntuku, Jacques Kouakou, Jane Achan, James Tibenderana, Jayne Webster, Joelle Sobngwi, Junior Voundi Voundi, Katherine Theiss-Nyland, Kone Fatoumata, Kwaku Poku Asante, Luís Mahumane, Mary Hamel, Paul Milligan, Peter Olumese, Rachel Belt, Rafiq Okine, Roly Gosling, Saliu Ogunmola, Shalom Tchokfe Ndoula, Therese Bleu, Valerie Makoge, Wilfred Mbacham, William Yavo

**Affiliations:** London School of Hygiene and Tropical Medicine, London, UK

**Keywords:** Malaria vaccine, Perennial Malaria Chemoprevention (PMC), Co-implementation, R21, RTS,S

## Abstract

Progress in malaria control will rely on deployment and effective targeting of combinations of interventions, including malaria vaccines and perennial malaria chemoprevention (PMC). Several countries with PMC programmes have introduced malaria vaccination into their essential programmes on immunizations, but empirical evidence on the impact of combining these two interventions and how best to co-implement them are lacking. At the American Society of Tropical Medicine and Hygiene 2023 annual meeting, a stakeholder meeting was convened to identify key policy, operational and research gaps for co-implementation of malaria vaccines and PMC. Participants from 11 endemic countries, including representatives from national malaria and immunization programmes, the World Health Organization, researchers, implementing organizations and funders attended. Identified evidence gaps were prioritized to select urgent issues to inform co-implementation. The output of these activities is a strategic roadmap of priority malaria vaccine and PMC co-implementation evidence gaps, and solutions to address them. The roadmap was presented to stakeholders for feedback at the 2024 Multilateral Initiative on Malaria meeting and revised accordingly. The roadmap outlines four key areas of work to address urgent evidence gaps for co-implementation: (1) support to the global and national policy process, (2) implementation support and research, (3) clinical studies, and (4) modelling. Together, these areas will provide practical guidance on the co-implementation of the interventions, and robust evidence to inform decision-making on how best to design, optimize and scale-up co-implementation in different contexts, including if and in what contexts the co-implementation is cost-effective, and the optimal schedule for co-implementation. This will work towards supporting the policy process on co-implementation of malaria vaccines and PMC, and achieving the most impactful use of available resources for the prevention of malaria in children.

## Background

Despite major progress in the fight against malaria over the past decades, progress in malaria control has stalled in sub-Saharan Africa, where over 90% of global cases and deaths occurred in 2023 [1]. Reducing the burden of malaria will depend on recognizing and responding to complex and diverse contexts with scarce resources to deliver an increasing number of available interventions efficiently and equitably. Progress will rely on scale-up of new and existing cost-effective interventions, including vector control, case management, chemoprevention and vaccines, and effective targeting of potential combinations of these partially effective interventions. These interventions include malaria vaccines, and perennial malaria chemoprevention (PMC).

The World Health Organization (WHO) recommends the use of two malaria vaccines, RTS,S/AS01_E_ (hereafter referred to as RTS,S) and R21/Matrix-M (hereafter referred to as R21), for the prevention of *Plasmodium falciparum* malaria in children living in areas with moderate to high transmission of malaria [2]. Both vaccines are currently being introduced sub-nationally and scaled up in 17 countries in sub-Saharan Africa; 26 countries have been approved for Gavi support, and additional countries have applied for Gavi support.

Despite an initial WHO recommendation in 2010, adoption and implementation of intermittent preventive treatment in infants (IPTi) with sulfadoxine-pyrimethamine (SP) has been very limited. The stalled progress in malaria control over recent years, combined with recent evidence, has led to a renewed interest in reactivating and improving the IPTi regimen and extending its use beyond infancy. In 2022, the WHO updated its guidelines for IPTi, allowing more flexibility in dosing regimens, age groups and drug choice, and renamed the intervention perennial malaria chemoprevention (PMC) [3].

**Fig. 1:**
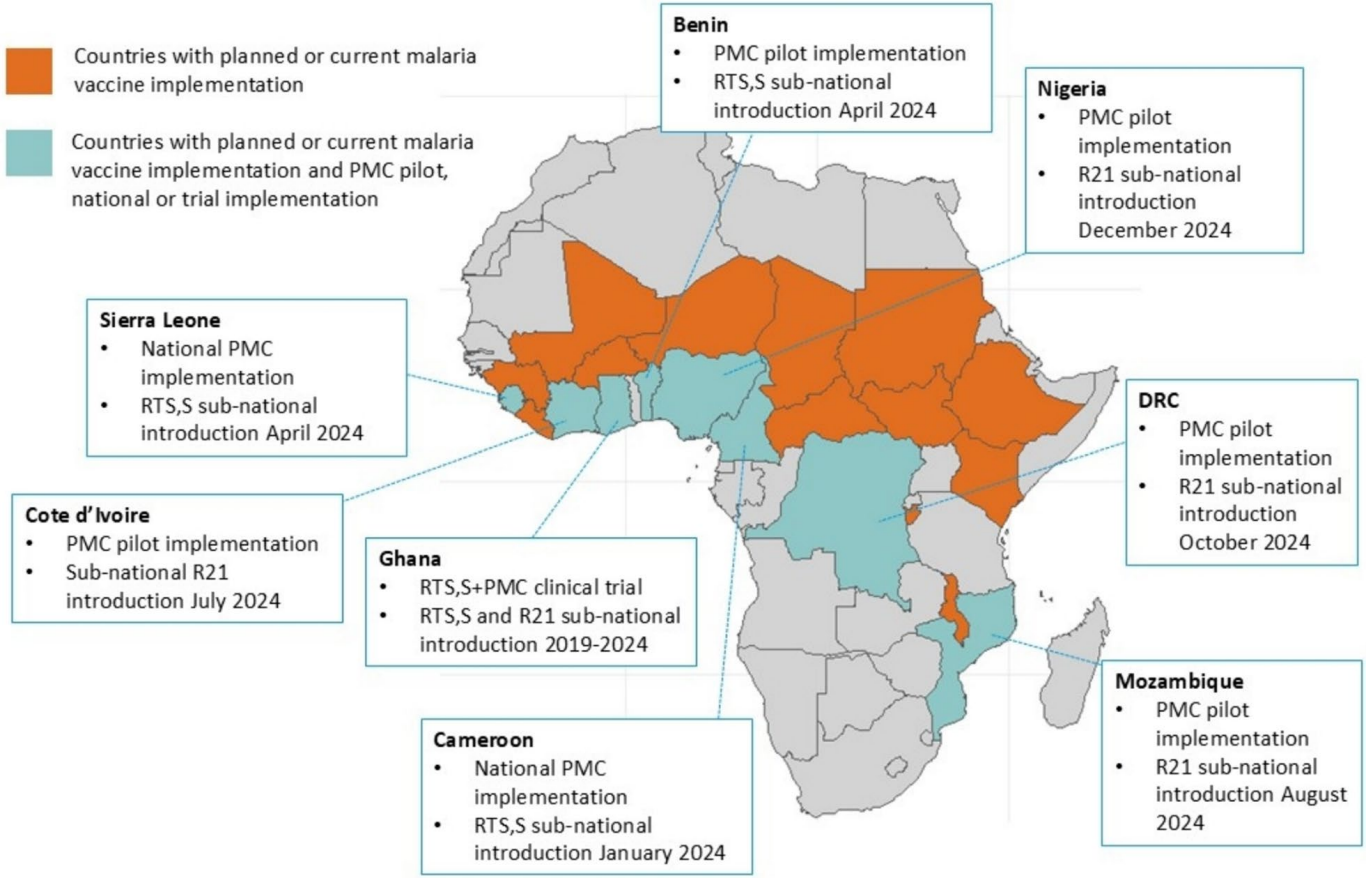
Overlap of malaria vaccination and PMC implementation

Both malaria vaccines and PMC target similar age ranges in young children and are delivered using the same healthcare contact points through the essential programme on immunization (EPI). EPI uses a variety of strategies to deliver vaccinations to children, including fixed, outreach and mobile strategies. Recently, alongside the increased flexibility in dosing regimens and age groups, additional delivery channels are being explored for PMC, including co-delivery with Vitamin A supplementation, and community delivery channels. Several countries with routine or pilot implementation of PMC, have recently introduced malaria vaccination into their EPI programme sub-nationally (Fig. 1); in Cameroon and Sierra Leone where PMC is being implemented routinely by the national programmes, the RTS,S vaccine is being routinely co-implemented with PMC. In other countries, including Benin, Côte d’Ivoire and DRC, malaria vaccine is also being co-implemented with PMC in districts with ongoing pilot PMC programmes. Additional countries with PMC pilot implementation have introduced malaria vaccines into different districts but may deploy co-implementation in the future.

**Fig. 2:**
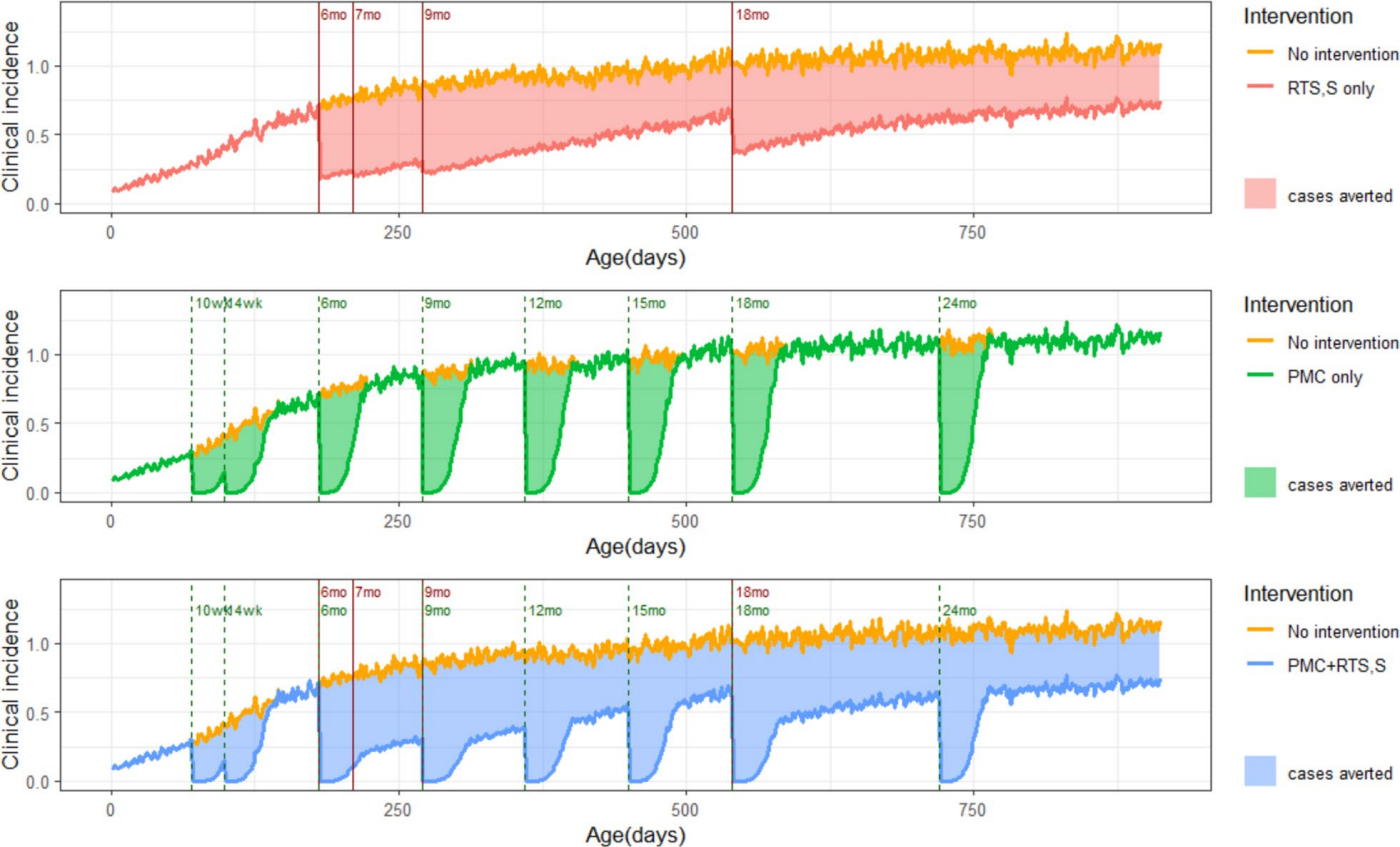
Modelled effects of combining RTS,S and PMC. Potential additive effects of combining RTS,S/AS01, PMC with eight doses of SP (A Mousa, I Atkinson, P Winskill and L Okell) using ‘malaria simulation’, Imperial College London, UK. RTS,S assumptions from White et al*.* [6]; PMC with SP providing 30 days protection with eight doses; coverage of all doses 100%

The R21 and RTS,S malaria vaccines are pre-erythrocytic vaccines, whilst malaria chemoprevention interventions such as PMC and seasonal malaria chemoprevention (SMC) target the blood stage of infection. Therefore, combining a pre-erythrocytic vaccine and a blood stage anti-malarial drug may have an additive or synergistic effect. The combination of seasonal malaria vaccination with the RTS,S vaccine and SMC when compared to SMC alone, reduced clinical and severe malaria, and malaria mortality by 60–70% in areas of highly seasonal transmission, during five years of follow-up [4]. Modelling suggests that combining malaria vaccine with PMC could also provide greater protection against malaria than either intervention given alone (Fig. 2) [5]. However, there is no empirical evidence on the effect of combining these two interventions, and many questions remain on how to co-implement the interventions in a way that optimizes resource use and maximizes impact.

## Objectives

In light of the introduction of malaria vaccines, including introduction in areas implementing PMC, and the evidence gaps on the combination of the interventions, a stakeholder meeting was held at the American Society of Tropical Medicine and Hygiene (ASTMH) 2023 annual meeting. The meeting outputs were processed and presented back to stakeholders at the 2024 Multilateral Initiative on Malaria (MIM) meeting. The objectives of the meeting and its follow-up were to:

1. Identify the key policy, operational and research gaps for the co-implementation of malaria vaccines and PMC.
2. Identify which of these gaps are an urgent priority to guide implementation and decision-making on co-implementing malaria vaccines and PMC.
3. Develop a roadmap of the priority gaps, with methods to address them, to support the cost-effective scale-up of these interventions and use of the available resources for the prevention of malaria in children.

## Evidence gaps for the co-implementation of malaria vaccines and PMC

### Stakeholder consultation to identify evidence gaps

Eighty-eight stakeholders participated in the two and a half hour meeting at ASTMH 2023, held in Chicago, USA, with representatives from 11 malaria endemic countries. Participants included representatives from: the National Malaria Programmes (NMPs) in Cameroon, Ghana, Côte d’Ivoire, Democratic Republic of Congo, Mozambique, Sierra Leone, and Zambia, and the EPI in Cameroon; the WHO offices in Geneva, the Africa Regional Office and Sierra Leone; the Fobang Institutes for Innovations in Science and Technology (FINISTECH), Cameroon; Ifakara Health Institute, Tanzania; Institut National de Sante Publique (INSP), Cote d’Ivoire; Institut Sciences et Techniques (INSTech), Burkina Faso; Institut de Recherche en Sciences de la Sante (IRSS), Burkina Faso; Kenya Medical Research Institute (KEMRI), Kenya; Kintampo Health Research Centre (KHRC), Ghana; London School of Hygiene and Tropical Medicine (LSHTM), UK; Malaria Research and Training Center (MRTC), Mali; Northwestern University, USA; Tulane University, USA; University of California San Francisco (UCSF), USA; University of Health and Allied Science, Ghana; University of Oxford, UK; Centers for Disease Control and Prevention (CDC), USA; Malaria Consortium, UK; PATH, USA; President's Malaria Initiative (PMI), USA; Population Services International (PSI) representatives from Benin, Cameroon, Côte d’Ivoire and Mozambique and USA offices; GlaxoSmithKline (GSK); GiveWell; UNITAID and U.S. Agency for International Development (USAID).

Roly Gosling (LSHTM, UK) introduced the meeting, providing an overview of the overlap in malaria vaccine roll-out and PMC, and the aims of the meeting, following which two brief presentations were given to set the context of the malaria vaccine roll-out and the current research being conducted on the combination of malaria vaccines and PMC. Firstly, Mary Hamel (WHO, Geneva) gave an overview of the WHO recommendations on malaria vaccination, the efficacy of the RTS,S and R21 vaccines, the roll-out of malaria vaccines, and the potential to achieve the highest reduction in malaria burden when combinations of malaria interventions are used strategically together. Following this presentation, Kwaku Poku Asante (KHRC, Ghana) presented an overview of the individually randomised controlled trial currently being conducted by KHRC and LSHTM that is investigating the efficacy of the RTS,S vaccine with and without PMC with either SP or SP-Amodiaquine (PACTR202307828402450). This study is funded by PMI, the Bill and Melinda Gates Foundation, and GiveWell via PMI Insights.

Following the presentations, participants took part in facilitated group discussions held in both English and French, with different groups formed to discuss each of the key (1) research, (2) operational, and (3) policy and strategic guidance gaps, challenges and opportunities for the co-implementation of malaria vaccines and PMC. These discussions used the World Café method, where the stakeholders participated in each of the discussion groups, with the first group brainstorming the key gaps, challenges, and opportunities in each of the three areas, which were then reflected and expanded on by the subsequent groups. The discussions were captured by notetakers and summarised in plenary at the end of the meeting.

The outcomes of these discussions on the questions/evidence gaps are presented in Tables 1, 2, 3. Implementation research gaps were combined with operational gaps in Table 2 to prevent repetition and overlap.

**Table 1:**
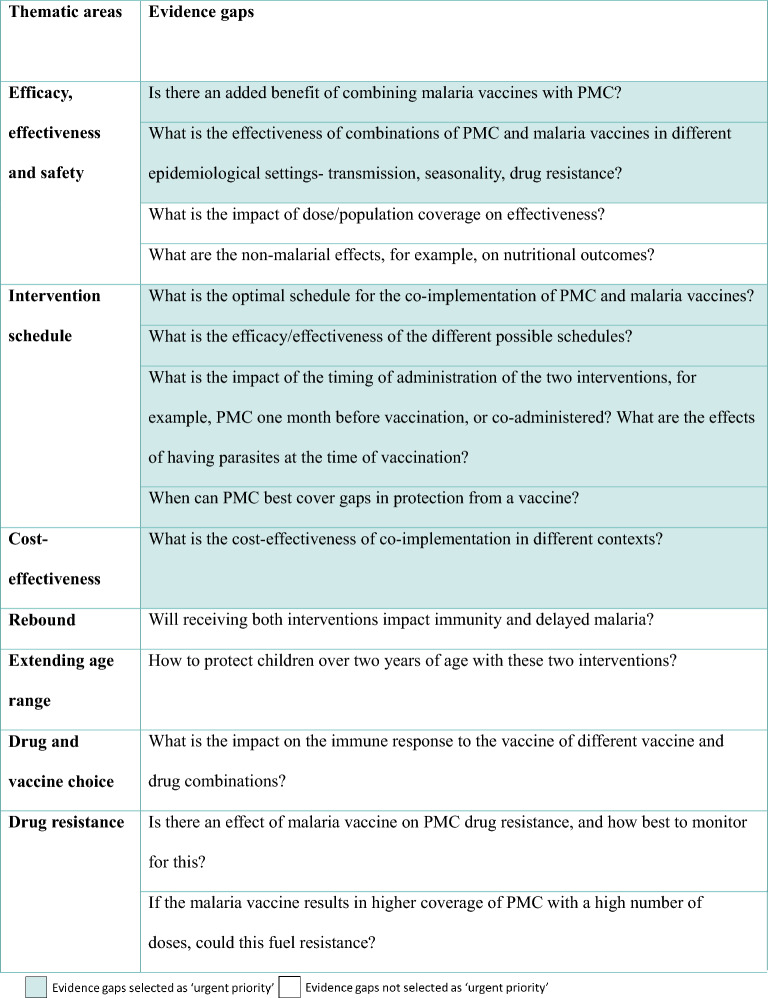
Research evidence gaps, highlighting those categorised as urgent priority

**Table 2:**
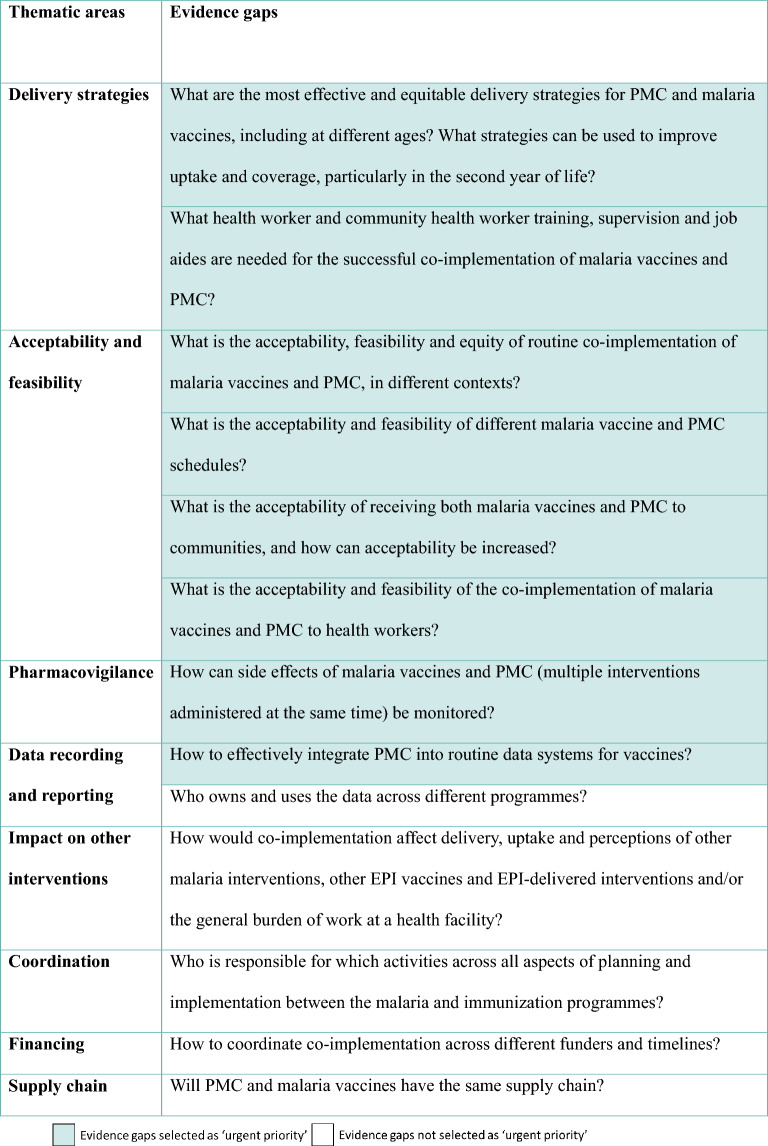
Implementation research and operational gaps, highlighting those categorised as urgent priority

**Table 3:**
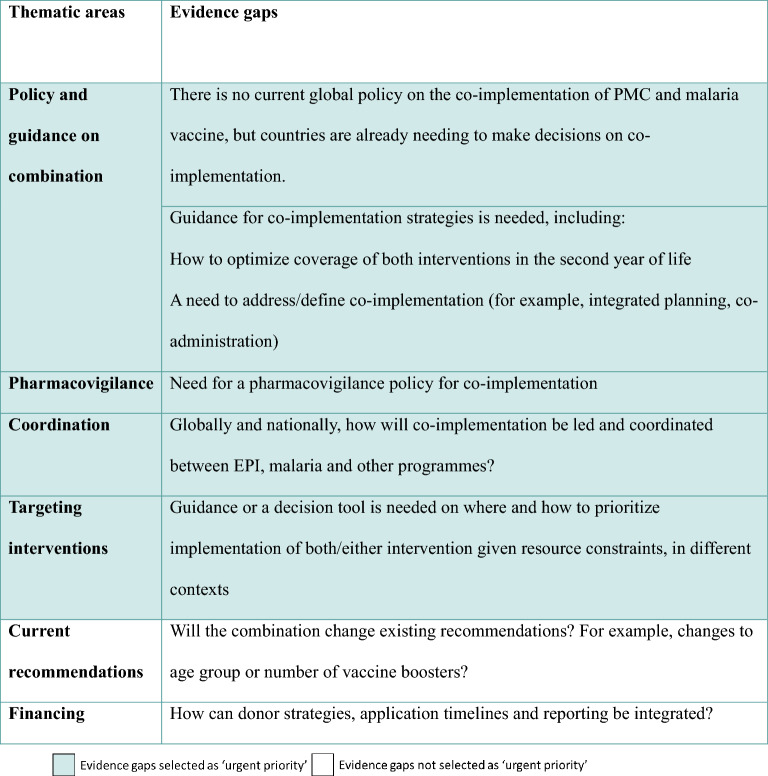
Policy and strategic guidance gaps, highlighting those categorised as urgent priority

### Prioritization of evidence gaps for the combination of malaria vaccines and PMC

Following the ASTMH meeting, research, operational, policy and strategic guidance gaps were organized into thematic areas and underwent an initial prioritization process to select the highest priority questions based on the needs of countries for their policy and strategy setting and implementation planning, the urgency of the questions needed to inform upcoming co-implementation, and the feasibility of addressing these questions. The initial prioritization was completed by three reviewers (Roly Gosling, LSHTM, Jane Grant, LSHTM and Henry Ntuku, PATH) and was subsequently validated by a prioritization survey on Microsoft Forms that was sent to the group of stakeholders who were invited to attend the meeting at ASTMH in 2023. The survey listed the questions identified at the ASTMH meeting and respondents were asked to select the priority level of each question as urgent priority, high/medium priority, or low/non-urgent priority. Urgent priority was defined as “need to know this today or in the near future to guide co-implementation of PMC and malaria vaccines and/or decision-making on whether to support co-implementation of PMC and malaria vaccination”. There was no limit on the number of urgent priority questions that respondents could select. Thirty-seven stakeholders responded to the survey, including 17 researchers, 9 representatives from implementing organizations, four representatives from NMPs, 3 representatives from EPIs, 2 from WHO and 2 funders.

The questions that the majority of the group selected as urgent priority are presented in Tables 1, 2, 3, and were taken forward to be included in the roadmap. The other questions may still be important or helpful to know at a later point, but were not classed as urgent in order to inform the current and upcoming co-implementation.

### Roadmap of priority evidence gaps for malaria vaccine and PMC co-implementation

The final prioritized questions were used to develop a roadmap of urgent malaria vaccine and PMC evidence gaps, potential methods to address them and outputs, with the overall focus on the need to act rapidly and efficiently to answer these questions.

Many of the evidence gaps fit under broader questions in the thematic areas, and additionally there was overlap, which allowed them to be consolidated into high level questions to be addressed by the same methods within the roadmap.

#### Feedback on the roadmap

An initial draft of the roadmap was presented to the stakeholders at the 2024 Multilateral Initiative on Malaria (MIM) meeting held in Kigali, Rwanda for feedback. Additionally, the roadmap was emailed to the stakeholders alongside a Microsoft Forms survey to provide feedback on each section of the roadmap.

The 90-min feedback meeting at MIM opened with a presentation by Mary Hamel (WHO, Geneva) on the deployment of malaria vaccines, including an overview of the updated WHO recommendation on malaria vaccines, the key findings from the RTS,S malaria vaccine implementation programme (MVIP), and the potential high impact of combining malaria vaccination with other interventions such as insecticide-treated nets and chemoprevention in both seasonal and perennial transmission areas. An update on planned malaria vaccine introductions and where these overlap with sites with PMC implementation was also presented. Shalom Tchokfe Ndoula (EPI, Cameroon) provided an update on the implementation of malaria vaccination and PMC in Cameroon, where RTS,S was introduced sub-nationally in January 2024, and is currently being co-implemented with PMC in 29 districts. Examples of how RTS,S vaccine and PMC implementation have been integrated in Cameroon were shared, including the coordination between the EPI and NMP, the communication strategy employed with a focus on intervention complementarity and synergy, and the reporting and recording systems. Ndoula highlighted that despite the current co-implementation in Cameroon, many key questions remain at the country level that are represented within the roadmap. Following the presentations, Roly Gosling and Jane Grant (LSHTM, UK) presented the outcomes of the ASMTH meeting and prioritization process, and the initial roadmap.

The initial roadmap was revised based on the feedback from participants at the MIM meeting and the online survey. The feedback included suggestions on the key methods that should be used to address the evidence gaps, what the outcomes of the roadmap should be, and on how the roadmap might be simplified.

**Fig. 3:**
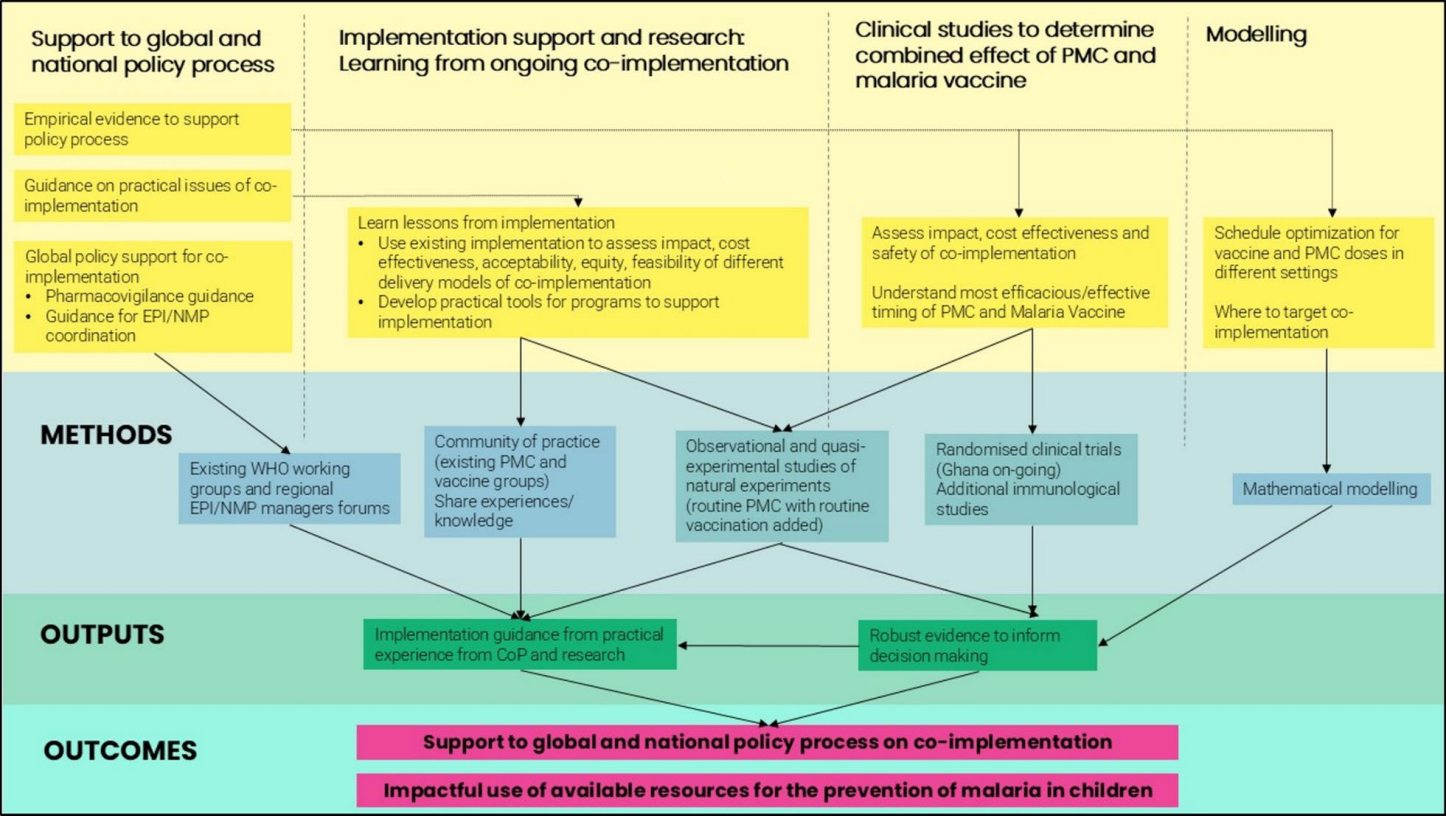
Malaria vaccine and PMC co-implementation priority evidence gaps roadmap

A schematic of the final roadmap is shown in Fig. 3. Four main areas were identified that should be addressed simultaneously: support to the global and national policy process, implementation support and research, clinical studies, and modelling. The roadmap is well-aligned with the broader malaria vaccine research agenda developed by WHO, GAVI, KHRC and PATH [7]. One of the six broad themes in the research agenda is the integration of the malaria vaccine with other interventions in seasonal and perennial settings, including PMC. This roadmap is a more detailed exploration of this theme within the broader malaria vaccine research agenda.

#### Support to global and national policy process

Two key priority policy and strategic guidance gaps in the roadmap included the need for empirical evidence on the combination of malaria vaccines and PMC, including evidence of the impact of the combination, to support the global guidance and national decision-making on if and how malaria programmes should co-deploy both interventions, and the need for guidance on practical issues of co-implementation. These two gaps are addressed by the other three areas of the roadmap. The remaining two policy and strategic guidance priority gaps identified, a need for global guidance on pharmacovigilance and EPI-NMP coordination for co-implementation, can be addressed internally within the WHO Global Malaria Programme and Immunization, Vaccines and Biologicals existing working groups, and regional EPI and NMP managers forums.

#### Implementation support and research: learning from ongoing co-implementation

The experiences of countries with current and upcoming malaria vaccine and PMC co-implementation (Fig. 1) can be used to learn programmatic lessons from the ongoing co-implementation and help in the development of practical tools to support co-implementation. These can include a guide on co-implementation, including details on delivery strategies for successful co-implementation, community engagement strategies, health worker training and job aids, pharmacovigilance and how to effectively adapt data recording and reporting systems. This knowledge and experience sharing can be done through a community of practice (CoP) on malaria vaccine and PMC co-implementation that draws upon the existing PMC and malaria vaccine groups, including the PMC CoP, Malaria Vaccine Coordination Team, EPI and NMP regional managers forum, and also through the Roll Back Malaria technical working groups. The CoP should also be expanded to include representatives from maternal and child health and nutrition programmes in order to facilitate cross-sector collaboration and a coordinated approach to child health. The lessons learned and best practices can be used to inform global guidance from the WHO.

Additionally, opportunistic observational and quasi-experimental studies can be conducted alongside the routine co-implementation, generating natural experiments in different contexts. These contexts could include differences in malaria transmission, SP resistance, strength of the EPI platform, malaria vaccine and PMC schedule used, delivery channels used, implementation of supportive strategies, and the vaccine used (R21 or RTS,S). Observational and quasi-experimental studies could include cohort studies, case–control and test negative case control studies, sentinel surveillance sites, qualitative studies and cross-sectional surveys. These studies can be used to assess the effectiveness, cost-effectiveness, coverage and equity of coverage, acceptability, and feasibility of co-implementation in these different settings. In particular, sites with ongoing PMC or vaccine research should take advantage of this opportunity to answer these questions with relatively small additional investments to measure impact and understand challenges to intervention success.

Together, experience of implementation and implementation research will provide the practical tools and guidance needed for current and new co-implementation as the strategies are scaled-up, and will contribute to the evidence base to inform global and national level decision-making on whether to continue co-implementation.

#### Clinical studies to determine synergy between PMC and malaria vaccines

Additional clinical studies are needed to assess impact and cost-effectiveness of co-implementation, and how to maximize impact of the combination of the two interventions by understanding the most efficacious/effective timing of the PMC and malaria vaccine dosing schedules. While quasi-experimental and observational studies will assess this in routine settings, randomized clinical trials are also needed to assess efficacy of the combination of the two interventions under trial conditions. One such trial is ongoing in the Bono East region in Ghana, as mentioned above (PACTR202307828402450). Additional trials may need to be conducted in other epidemiological settings, including those with different levels of SP resistance and malaria transmission, to determine under what settings a cost-effective impact is seen in adding PMC to malaria vaccination. Additionally, studies are needed to determine the immune response to the vaccine when it is co-administered with PMC.

#### Modelling

Mathematical modelling should utilize existing and emerging data from the clinical, observational and quasi-experimental studies to understand how to optimize the schedule for malaria vaccine and PMC doses in different settings, and the most effective way to target implementation of the two interventions and their combination alongside other malaria interventions in different contexts, given resources constraints. Modelled findings should be validated by empirical data when opportunities arise.

## Conclusion

The roadmap outlines the four areas of work needed to swiftly and efficiently address the urgent evidence gaps for malaria vaccine and PMC co-implementation that were identified by a diverse group of key stakeholders: support to the global and national policy process, implementation support and research, clinical studies, and modelling. The sharing of practical experiences of co-implementation and implementation research will provide the guidance needed on co-implementation of malaria vaccines and PMC to support the ongoing and forthcoming co-implementation in several countries in sub-Saharan Africa. The implementation and clinical research and modelling work will provide robust evidence needed to inform decision-making on whether to continue and scale-up the co-implementation and in which contexts this is cost-effective. The four areas contribute towards the ultimate outcomes of supporting the policy process on co-implementation for malaria vaccines and PMC, and achieving the most impactful and equitable use of available resources for prevention of malaria in children.

We hope that partners, from funders to implementers, can use this roadmap to ensure that evidence gaps are filled rapidly, help to focus resources on relevant research, and support coordinated action in support of malaria vaccine and PMC scale up. The roadmap will be a living document and will periodically be updated as research findings and implementation experience provide further refinement.

## Data Availability

No datasets were generated or analysed during the current study.

## Abbreviations

ASTMH: American Society of Tropical Medicine and Hygiene
CoP: Community of Practice
EPI: Essential Programme on Immunization
IPTi: Intermittent Preventive Treatment in infants
KHRC: Kintampo Health Research Centre
LSHTM: London School of Hygiene and Tropical Medicine
MIM: Multilateral Initiative on Malaria
NMP: National Malaria Programme
PMC: Perennial Malaria Chemoprevention
SMC: Seasonal Malaria Chemoprevention
SP: Sulfadoxine-Pyrimethamine
WHO: World Health Organization
