# Correction: A roadmap of priority evidence gaps for the co-implementation of malaria vaccines and perennial malaria chemoprevention

**DOI:** 10.1186/s12936-025-05410-w

**Published:** 2025-06-24

**Authors:** Jane Grant, Perennial Malaria Chemoprevention (PMC) community of practice sub-group on the co-implementation of malaria vaccines and PMC

**Affiliations:** London School of Hygiene and Tropical Medicine, London, UK

**Correction: Malaria Journal (2025) 24:126** 10.1186/s12936-025-05347-0

Following publication of the original article, it came to the journal's attention that the name of the group authorship (which is listed after the first author) was incomplete: 'and PMC' had been omitted from the end of the name. The author list has since been corrected in the published article [1]. The authors thank you for reading this erratum and apologize for any inconvenience caused.
